# Use of Alginates as Food Packaging Materials

**DOI:** 10.3390/foods9101440

**Published:** 2020-10-12

**Authors:** Michael G. Kontominas

**Affiliations:** Laboratory of Food Chemistry, Department of Chemistry, University of Ioannina, 45110 Ioannina, Greece; mkontomi@uoi.gr; Tel.: +30-26510-08342

**Keywords:** food packaging, films, coatings, alginates, preservation, quality retention

## Abstract

Packaging mainly functions by protecting and preserving its contents. In the case of food packaging, the package protects the contained food product from (i) physical/mechanical damage; (ii) physico-chemical changes due to the effect of light, oxygen, moisture and odors; and (iii) biological changes due to the presence of microorganisms and pests; all the above parameters result in the reduction in product quality and safety. Due to the negative impact of synthetic packaging materials on the environment, research organizations as well as the food industry are currently exploring the possibility of using biodegradable and renewable materials deriving from natural sources. Such biopolymers include: proteins (whey proteins, wheat, corn and soy proteins, gelatin), lipid derivatives (waxes, acetylated triglycerides) and carbohydrates (starch, cellulose and its derivatives, carrageenan, pectin, chitosan, alginates) used in food packaging applications. Alginates are natural hydrophilic polysaccharide biopolymers mainly extracted from marine brown algae. In the form of films or coatings, they exhibit: good film-forming properties, low permeability to O_2_ and vapors, flexibility, water solubility and gloss while being tasteless and odorless. When combined with additives such as organic acids, essential oils, plant extracts, bacteriocins and nanomaterials, they contribute to the retention of moisture, reduction in shrinkage, retardation of oxidation, inhibition of color and texture degradation, reduction in microbial load, enhancement of sensory acceptability and minimization of cooking losses. Alginates were initially used as a coating for perishable fresh fruits and vegetables to control respiration rate, but can be applied to a wide range of foods, such as meat, poultry, seafood and cheese products, resulting in the extension of product shelf life. When used as part of the principle of active, intelligent and green packaging technologies, alginates can work synergistically to yield a multi-function food packaging system comprising the ultimate goal of food packaging technology.

According to the former Packaging Institute International [1], packaging is defined as the enclosure of products or items in a wrapped pouch, bag, box, cup, tray, can, tube, bottle or other container form to perform one or more of the following functions: containment, protection, preservation, communication, utility and performance. Packaging becomes substantially more critical when it comes to food, pharmaceutical and medicinal products that come into contact with human internal organs, thus directly affecting consumer wellbeing. Among the above functions of packaging, protection and preservation are of the utmost importance. The package protects the contained food product from (i) physical/mechanical damage, (ii) physico-chemical and (iii) biological changes which result in the reduction in product quality and safety [2]. The preservation function of packaging is the result of the passive barrier it creates between the food and the surrounding atmosphere with regard to the effect of light, oxygen, moisture, odors and biological contamination by spoilage and pathogenic microorganisms and pests. With the advancement of technology, packaging has evolved from a simple passive barrier to an interactive system between the contained product and the packaging material, leading to technologies known as Modified Atmosphere Packaging, Vacuum Packaging, Active Packaging, Intelligent Packaging and Green Packaging.

Conventional petroleum-derived plastics, currently used in food packaging applications, are considered as ”environmentally unfriendly materials” and are responsible for huge amounts of waste ending up in the marine and land ecosystem directly affecting marine and wildlife [3].

To tackle the environmental pollution problem, scientists have begun to explore the possibility of using natural, biodegradable and renewable packaging materials for food packaging applications. The EU has adopted the strategy of bioeconomy in order to protect the environment and the wellbeing of its citizens. According to these plans, by 2030, all packaging on the EU market will be recyclable, and the consumption of plastics will be substantially reduced. The EU is committed to reducing plastic waste, stopping mass storage and investing in natural and innovative packaging materials [4].

Such biomaterials are produced from natural sources and include proteins (whey proteins, wheat, corn and soy proteins, gelatin), lipid derivatives (waxes, acetylated triglycerides) and carbohydrates (starch, cellulose and its derivatives, carrageenan, pectin, chitosan, alginates) [5,6,7]. Among these biopolymers, alginates in the form of films and coatings exhibit good film-forming properties, low permeability to O_2_ and vapors, flexibility, good tensile strength, flexibility, tear resistance, rigidity, water solubility and gloss while being tasteless and odorless. When combined with additives such as essential oils, plant extracts, bacteriocins, enzymes, chitosan, organic acids, metallic nanoparticles and chelating agents, they contribute to the retention of moisture, reduction in shrinkage, retardation of oxidation, color and texture degradation, reduction in microbial counts, improvement of mechanical and barrier properties, enhancement of sensory acceptability and minimization of cooking losses [8,9].

Alginates are natural polymers isolated from the cell walls of brown algae (*Phaeophyceae*) where they are found in the form of sodium, calcium and magnesium salts of alginic acid; they can also be synthesized by certain bacteria (*Pseudomonas* and *Azotobacter*) [10]. They are linear copolymers with blocks of (1→4)-α-l-guluronic acid (G), (1→4)-β-d-mannuronic acid blocks (M) and heteropolymeric sequences of M and G (MG blocks). The M/G ratio as well as the length of each block can be estimated through proton NMR spectroscopy. The different combinations between M and G blocks lead to the manufacture of at least 200 different alginates [11]. The FDA (US Food and Drug Administration) has recognized alginates as GRAS (generally recognized as safe) classified substances [12], and the EFSA (European Food Safety Authority) has authorized the use of alginate and related salts in specific doses [4]. Commercially, alginates are mainly available as sodium alginate, commonly used as a thickener, stabilizer and gelling agent in food such as deserts, sauces, stabilizers and beverages [13].

A major drawback of alginates is their high water vapor transmission rate owing to their high hydrophilicity. The poor moisture barrier of alginate packaging is due to swelling caused after absorption of moisture vapor from the surroundings that increases the water vapor transmission and water uptake by the packaging material [14]. This problem has been tackled by incorporating Montmorillonite/cellulose nanoparticles or Montmorillonite clay into the alginate matrix [14]. In the form of Active Packaging, the addition of (i) polylactic acid has been used to increase oxygen barrier properties; (ii) CaCl_2_ has been used for the improvement of mechanical properties, resistance to water and water vapor transmission rate; (iii) glycerol and sorbitol have been used for increasing polymer flexibility; (iv) lipids such as corn oil and olive oil have been used to minimize water loss; (v) microfibrillated cellulose has been used to enhance mechanical properties; (vi) nisin has been used to suppress gram(+) bacterial growth; (vii) potassium sorbate, essential oils (lemongrass, oregano, thyme, rosemary, cinnamon, garlic, etc.) and silver-montmorillonite nanoparticles have been used as antimicrobials to extend product shelf life; (viii) essential oils have been used as antioxidants to retard lipid oxidation; (ix) lactic acid bacteria have been used for the formation of biofilms to control the growth of food-borne pathogens in ready-to-eat food; (x) organic acids have been used to protect muscle foods by inhibiting growth of food pathogens such as *L. monocytogenes*, *Salmonella typhimurium* and *Escherichia coli* (*E. coli.*) [8,14].

Alginate salts both as packaging films and coatings have been used for preservation purposes in a large variety of food commodities in the literature: for shelf life extension of potato strips and fresh-cut apples [15,16]; for the reduction in browning of fresh-cut mangoes during storage [17]; for the retention of sensory quality of mushrooms [18] and fresh-cut carrots [19] in combination with nanoparticles; for shelf life extension of pineapple in combination with modified atmosphere packaging [20] for quality retention and antioxidant properties of sweet cherries [21]; for shelf life extension of peaches [22] and melons in combination with essential oils [23]; for the prevention of dehydration and retention of sensory characteristics of pork patties [24]; for the retention of color, prevention of drip loss and off-odor detection of beef steaks in combination with HOCl [25]; to inhibit the growth of pathogenic bacteria such as *L. monocytogenes*, *Salmonella typhimurium* and *E. coli* [26]; for the improvement of microbial quality of chicken meat in combination with nisin and essential oils [27]; for the improvement of beef consumer acceptability in combination with essential oils [28]; to retain quality and extend shelf life of fresh pork in combination with carboxymethyl cellulose and epigallocatechin [29]; to control the growth of *L. monocytogenes* in ham slices in combination with oregano essential oil [30]; to improve quality and extend shelf life of chicken nuggets in combination with green tea extract [31]; to control *L. monocytogenes* in sliced cooked ham in combination with vacuum packaging and the addition of enterocins [32]; to prevent moisture loss and lipid oxidation in precooked ground-beef patties in combination with α-tocopherol and vacuum packaging [33]; to inhibit *Staphylococcus aureus* in beef in combination with nisin [34]; to inhibit *L. monocytogenes* in smoked salmon in combination with lactic acid bacteria [35]; to retard microbial growth and chemical changes in rainbow trout with the addition of resveratrol [36]; to enhance quality by retarding lipid oxidation in sea bass in combination with smoking and the addition of resveratrol [37]; to preserve quality and extend shelf life of red sea bream with the addition of 6-gingerol [38]; to preserve the quality and inhibit *Escherichia coli* O157:H7 of silver carp fillets in combination with carboxymethyl cellulose and clove essential oil [39]; to extend the shelf life of Mozzarella cheese in combination with Modified atmosphere packaging [40], etc.

In conclusion, when used as part of the principles of active, intelligent and green packaging technologies, alginates can work synergistically to yield a multi-function food-packaging system comprising the ultimate goal of modern food packaging technology.

The present manuscript is an editorial for the recently published article in the journal FOODS entitled: ”Alginate-Based Edible Films and Coatings for Food Packaging Applications” authored by Parreidt et al., 2018, comprising a comprehensive presentation of the fundamentals of alginate-based films and coatings as well as their detailed application to the packaging of specific foods.
